# The Spectrum of Minimal Change Disease/Focal Segmental Glomerulosclerosis: From Pathogenesis to Proteomic Biomarker Research

**DOI:** 10.3390/ijms26062450

**Published:** 2025-03-09

**Authors:** Yuriy Maslyennikov, Andrada Alina Bărar, Crina Claudia Rusu, Alina Ramona Potra, Dacian Tirinescu, Maria Ticala, Alexandra Urs, Ioana Ecaterina Pralea, Cristina Adela Iuga, Diana Tania Moldovan, Ina Maria Kacso

**Affiliations:** 1 Department of Nephrology, Faculty of Medicine, “Iuliu Hațieganu” University of Medicine and Pharmacy, 400012 Cluj-Napoca, Romania; maslyennikov_yuriy@elearn.umfcluj.ro (Y.M.); barar_andrada_alina@elearn.umfcluj.ro (A.A.B.); claudia.rusu@umfcluj.ro (C.C.R.); alina.potra@umfcluj.ro (A.R.P.); tirinescu.dacian@umfcluj.ro (D.T.); cosa.maria@umfcluj.ro (M.T.); alexandra.urs@elearn.umfcluj.ro (A.U.); maria.kacso@umfcluj.ro (I.M.K.); 2 Department of Personalized Medicine and Rare Diseases, MedFuture—Research Centre for Biomedical Research, “Iuliu Haţieganu” University of Medicine and Pharmacy, 400349 Cluj-Napoca, Romania; pralea.ioana@umfcluj.ro (I.E.P.); cristina.iuga@medfuture.ro (C.A.I.); 3 Department of Drug Analysis, Faculty of Pharmacy, “Iuliu Hațieganu” University of Medicine and Pharmacy, 400349 Cluj-Napoca, Romania

**Keywords:** proteomics, minimal change disease, focal segmental glomerulosclerosis, podocytopathies, biomarkers

## Abstract

Podocyte injury plays a central role in both focal segmental glomerulosclerosis (FSGS) and minimal change disease (MCD). Pathogenic mechanisms are diverse and incompletely understood, partially overlap between FSGS and MCD, and are not reflected by kidney biopsy. In order to optimize the current variable response to treatment, personalized management should rely on pathogenesis. One promising approach involves identifying biomarkers associated with specific pathogenic pathways. With the advancement of technology, proteomic studies could be a valuable tool to improve knowledge in this area and define valid biomarkers, as they have in other areas of glomerular disease. This work attempts to cover and discuss the main mechanisms of podocyte injury, followed by a review of the recent literature on proteomic biomarker studies in podocytopathies. Most of these studies have been conducted on biofluids, while tissue proteomic studies applied to podocytopathies remain limited. While we recognize the importance of non-invasive biofluid biomarkers, we propose a sequential approach for their development: tissue proteomics could first identify proteins with increased expression that may reflect underlying disease mechanisms; subsequently, the validation of these proteins in urine or plasma could pave the way to a diagnostic and prognostic biomarker-based approach.

## 1. Background

While genomics has provided critical insights into the genetic foundations of diseases, proteomics, supported by recent technological advancements, offers a more immediate and dynamic understanding of disease mechanisms, biomarker identification, and treatment responses. This capability is particularly valuable in the context of personalized medicine, where tailored treatment strategies depend on a comprehensive understanding of an individual’s molecular profile. As such, proteomics serves as a vital complement to genomics in advancing modern pathology.

In the past two decades, in some glomerular diseases, tissue proteomics have clarified pathogenic mechanisms and led to the development of diagnostic and prognostic biofluid biomarkers promptly embedded in clinical practice [1]. In an area with an urgent need for pathogenic clarification and reclassification, such as minimal change disease (MCD) and focal segmental glomerulosclerosis (FSGS), proteomic studies have the potential to make a significant impact.

## 2. Podocyte Injury in the Continuum of MCD/FSGS

Minimal change disease and FSGS are both podocyte disorders primarily characterized by nephrotic syndrome associated with various degrees of foot process effacement (FPE) observed on electron microscopy (EM). The key distinction lies in light microscopy (LM) where FSGS presents with patterns of focal and segmental sclerosis while LM is typically normal in MCD. Additionally, MCD is associated with negative immunofluorescence staining, whereas in FSGS, low intensity complement deposition and/or IgM may be observed in the sclerotic areas, traditionally regarded as non-specific (trapping) [2,3]. From a clinical and therapeutic perspective, current guidelines suggest a classification of FSGS into primary, genetic, secondary and FSGS of undetermined cause [4]. Primary FSGS is characterized by diffuse FPE (generally over 80%) nephrotic syndrome; a specific cause cannot usually be identified. Secondary FSGS mostly presents with important proteinuria, possibly in the nephrotic range but without overt hypoalbuminemia; it has less abundant FPE (25–40%) and is usually due to adaptive mechanisms to focal podocyte injury. In genetic causes of FSGS, the degree of FPE is variable, from extensive in certain monogenic causes (e.g., NPHS 1) to focal in other instances (e.g., ACTN4 mutation that cause cytoskeletal structural changes that decrease podocyte resistance to injury or mechanical stress). FSGS of undetermined cause refers to forms without extensive FPE but without an identifiable secondary or genetic cause [5,6]. From a clinical point of view, it is important to distinguish between primary FSGS (amenable to immunosuppression) and secondary forms, treated conservatively. However, it is now widely recognized that this morphology-based classification is insufficient and does not fully capture the complexity of podocyte diseases.

It is generally accepted that FSGS is a pattern of response to various etiopathogenic factors, but it becomes increasingly clear that MCD, traditionally viewed as a distinct disorder, may also be included in this spectrum of diseases with different triggers and patterns of response. In fact, a unifying approach has been considered for the MCD/FSGS spectrum [2]. This perspective is reinforced by evidence that, in certain cases, progression from MCD to FSGS has been documented, with sampling error and the earlier stage of the disease potentially explaining an increased likelihood for observing an MCD pattern [2,7]. However, the most compelling argument for considering these diseases together lies in the shared podocyte injury and its direct consequences, namely, FPE and proteinuria.

A podocyte is a highly specialized and ontogenetically conserved cell with a primordial role in maintaining permselectivity of the glomerular filtration. Tertiary interdigitating foot processes (FPs) from neighboring podocytes are linked through a highly specialized protein structure—the slit diaphragm (SD). In its structure, several proteins form a ladder-like structure, which plays a crucial role in the glomerular filtration process by preventing protein loss. Additionally, podocytes are covered with anion charge, assured by the glycocalyx of the cells, which enhances further the filtration barrier [5,8].

The stability of a podocyte is assured through its anchoring to the glomerular basement membrane (GBM) via a specialized molecular assembly which is called focal adhesion (FA). These are intricate protein structures which link extracellular matrix (ECM) and GBM to the podocyte cytoskeleton. FA includes integrin as one of the most important transmembrane anchoring proteins [9]; importantly, FAs, like SD, do not just act as a mechanical anchor but also as a signaling hub, mediating the podocyte response to various stimuli. They play a critical role in regulating cell behavior and motility [9,10], influencing processes like cell migration survival, which are important for maintaining glomerular integrity and function.

Podocyte function is highly dependent on the integrity of its cytoskeleton that is mainly composed of actin fibers in the foot processes and of microtubules in the cellular body. The organization of the actin cytoskeleton is fine-tuned by proteins that control the polymerization and depolymerization of actin filaments as well as proteins that link these filaments into bundles or networks—referred to as actin-binding proteins. Key regulatory molecules, including RHO-GTPases such as Rac1, Rho A, and Cdc42, as well as other intracellular pathways, are responsible for regulating the actin cytoskeleton dynamics by interacting with signals from the SD and FA [9,10].

Noteworthy, podocytes are highly specialized and differentiated cells that do not divide. Lethal or sublethal injury to podocytes results in exposed areas of GBM, leading to the disruption of filtration barrier, proteinuria, and potentially subsequent inflammatory and profibrotic changes.

As a direct consequence of structural and functional alterations within podocytes and their actin cytoskeletons, the interdigitating structure of the FP becomes distorted, leading to FPE and compromising the permselectivity of the glomerular filtration barrier, resulting in proteinuria. However, the mechanisms underlying these morphological and functional changes differ significantly, both from an etiologic point of view and from a pathogenetic perspective.

**Figure 1:**
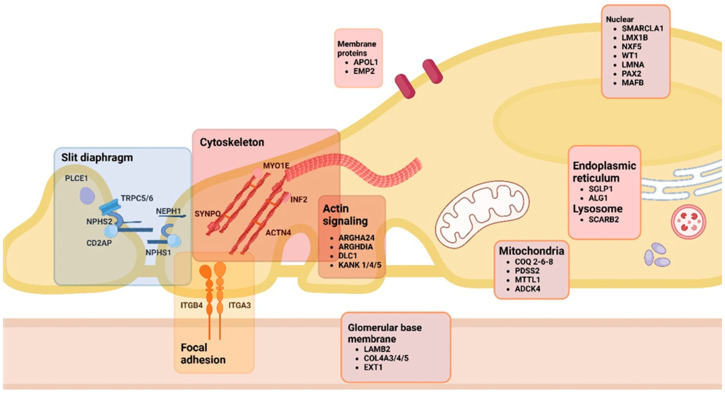
Schematic representation of podocyte pathogenic genes. Gene variants are grouped according to cellular structure. PLCE1, phospholipase C epsilon 1; TRPC 5/6, transient receptor potential channel 5/6; NPHS1, nephrin; NPHS2, podocin; CD2AP, CD2-associated protein; NEPH1, Kin of IRRE-like protein 1; MYO1E, myosin 1E; SYNPO, synaptopodin; INF2, inverted-formin2; ACTN4, alpha actinin 4; ITGB4, integrin ß4; ITGA3, integrin α3; ARGHA24, Rho-GTPase-activating protein 24; ARGHDIA, Rho-GDP-dissociation inhibitor 1; DLC1, Rho GTPase-activating protein 7; KANK 1/4/5, KN motif and ankyrin repeat domains 1/4/5; LAMB2, laminin subunit beta 2; COL4A3/4/5, collagen type IV aplha 3/4/5 chain; APOL1, Apolipoprotein A1; EMP2, epithelial membrane protein2; COQ 2/6/8, coenzime Q2/6/8; PDSS2, decaprenyl-diphosphate synthase subunit 2; MTTL1, mitochondrially encoded tRNA leucine 1; ADCK4, coenzyme Q8B; SGLP1, sphingosine-1-phosphate lyase 1; ALG1, chitobiosyldiphosphodolichol beta-mannosyltransferase; SCARB2, lysosomal integral membrane protein-2; SMARCLA1, SWI/SNF-related matrix-associated actin-dependent regulator of chromatin subfamily A-like protein 1; LMX1B, LIM homeobox transcription factor 1-beta; NXF5, nuclear RNA Export Factor 5; WT1, Wilms tumor 1; LMNA, lamin A/C; PAX2, paired box gene 2; MAFB, transcriptor factor MafB. This figure was created with BioRender.com.

**Figure 2:**
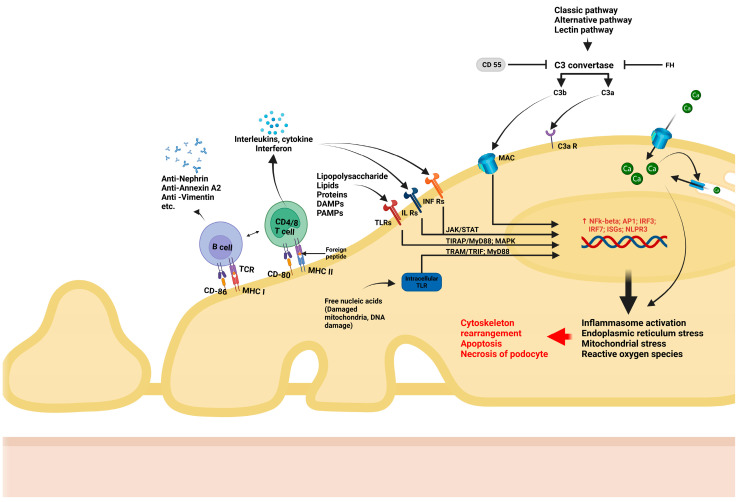
Schematic representation of immune-mediated podocyte injury. Podocytes interact with immune cells through major histocompatibility complex type I (MHC I) and II (MHC II) but also possess co-stimulatory molecules for B lymphocytes (CD86) and for T lymphocytes (CD80). Imbalance between effector and regulatory T lymphocytes, interleukin/cytokine synthesis, and B cell-mediated antibody synthesis against podocyte structures can occur due to podocyte interaction with immune cells. Toll-like receptors (TLRs) can detect signals like pathogen-related molecular patterns (PAMPs) and damage-related molecular pattern (DAMPs) both from outside (e.g., lipopolysaccharide, lipids, and proteins) and within the cell (e.g., free nucleic acids). Upon binding TLRs, increased transcription of inflammatory genes (NF-kß, IRF3, IRF7, and AP1) and inflammasome activation are mediated through intracellular signaling proteins such as TIRAP, MyD88 and MAPK, ultimately resulting in cytoskeleton rearrangement and apoptosis. Interleukin receptors (IL Rs) and interferon receptors (INF Rs) also increase the transcription of inflammatory and profibrotic factors through the JAK/STAT signaling pathway. Complement-mediated podocyte injury can occur due to sub-lytic amounts of membrane attack complex (MAC). The activation of the NF-kß pathway and a rapid increase in intracellular Ca^2+^ induce NFk-ß-mediated inflammatory response, mitochondrial stress, oxidative stress, and endoplasmic reticulum stress. The lack of inhibitor factors such as CD55 or complement factor H (FH) can trigger and intensify MAC production. This figure was created with BioRender.com.

**Figure 3:**
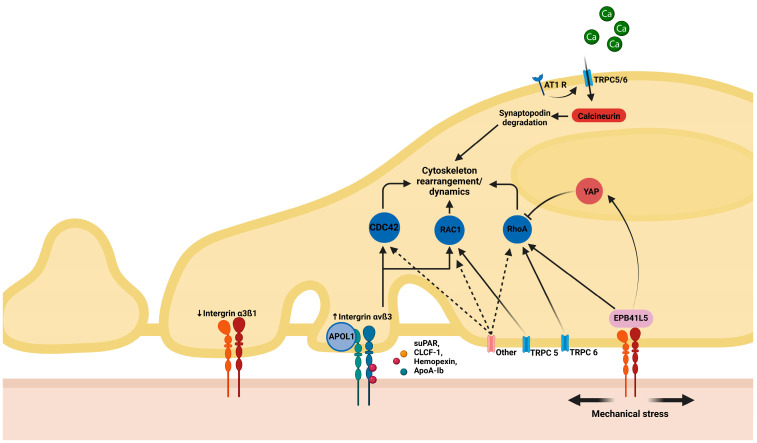
Schematic representation of mechanical stress-related podocyte injury. Angiotensin II receptor 1 (AT1 R) upon stimulation causes increased intracellular calcium influx through transient receptor potential channel 6 (TRPC6), followed by increased calcineurin activity and increased degradation of synaptopodin, an actin-binding protein. Cytoskeletal rearrangements are controlled by Rho GTPases: cell division control protein 42 (CDC42), Rac family small GTPase 1 (RAC1), and Ras homolog family member A (RhoA). Mechanical stress is sensed and transmitted by B integrins to erythrocyte membrane protein band 4.1 like 5 (EPB41L5). EPB41L5 activates intracellular pathways which regulate the translation of proteins that influence RhoA activity and cytoskeletal rearrangement. Yes-associated protein (YAP) is a transcription co-regulator factor stimulated by EPBB41L5 activity. YAP nuclear translocation results in the translation of proteins that regulate podocyte Rho GTPase activity. Membrane proteins like TRPC5/6 also can directly regulate the activity of Rho GTPases, other membrane proteins may be also involved in Rho GTPase regulation (dashed arrows). Permeability factors (soluble urokinase plasminogen activator receptor, suPAR; hemopexin, HPX; and cardiotrophin-like cytokine factor 1, CLCF1) interfere with FA; in particular, suPAR binds and activates αvß3 B integrin, further modulating cytoskeleton rearrangements via CDC42 and RAC1. APOL1 expression increases the linking of suPAR. This figure was created with BioRender.com.

First, an important differentiation is to be made regarding cytoskeleton rearrangement and subsequent FPE as a reactive process or as the initial culprit in podocyte disease [11,12,13]. In the first scenario, when there is focal (limited and not widespread) injury to the podocytes, there is an attempt to limit the degree of damage by structural and functional changes in the affected and neighboring cells that occur as an adaptative process. This might be the case in hyperfiltration-induced podocyte damage when FPE is the price paid for strengthened attachment at the level of FA; in this case, the podocyte sacrifices permselectivity in order to prevent further podocyte loss by improving adherence. On the other hand, in the second scenario, extensive and diffuse structural and functional changes in the cytoskeleton are the initial cause of FPE and proteinuria, resulting from genetic disease, antibody- or other immune-mediated injury, or yet unknown causes; in this case, the etiology and mechanisms are also very different [11,13,14]. The key pathways involved in this process are listed below and depicted in Figure 1, Figure 2 and Figure 3, which detail the intricate molecular interactions that contribute to podocyte dysfunction in the scenarios mentioned above.

### 2.1. Structural Deficiencies

Structural deficiencies of the SD, actin cytoskeleton, and FA can be the cause of podocyte dysfunction. Currently, more than 70 monogenic causes of proteinuric kidney disease have been identified, many of which comprise proteins of the SD structure (such as NEPH1, NPHS1, and NPHS2), as well as some proteins involved in the regulation of the actin cytoskeleton (ACTN4, INF2, etc.). Additionally, defects in myosin proteins and Rho-GTPase signaling pathways have been linked to podocyte dysfunction. Moreover, variants in genes encoding structures from GBM and ECM, like laminins and collagen, can lead to the dysfunction of filtration processes [15,16]. Podocyte pathogenic variants are depicted in Figure 1.

### 2.2. Virus-Induced Lesions

Other potential drivers of podocyte injury are virus-induced factors. Studies have demonstrated that podocytes possess sensors for double-stranded RNA, a byproduct of viral replication, and can lead to an innate response. This response includes an increase in the expression of toll-like receptors (TLRs) and other signaling molecules, resulting in the altering of podocyte SD structures including proteins such as NPHS1, NPHS2, and CD2AP. These changes, in turn, promote increased cytokine production and inflammation via the NF-κB pathway [17]. Investigations in murine models showed that interferon beta, a major component of antiviral response, can lead to podocyte loss [18]. In the context of HIV-associated nephropathy, it was shown that viruses are present in podocytes, directly damaging the actin cytoskeleton and contributing to podocyte dysfunction [19].

Sometimes, these mechanisms can act in concert or could be additive. For example, the two distinct polymorphisms in the apolipoprotein L1 (APOL1) gene (G1 and G2) that are frequent in West African patients and have monoallelic or biallelic inheritance (e.g., G1/G1, G1/G2, or G2/G2) are risk factors for FSGS, with the highest risk in people presenting two risk variants—some studies suggesting an up to 17-fold increase in risk [20,21]. However, a “second podocyte hit”, such as HIV-associated viral infection, increases this risk even more [22].

### 2.3. Permeability Factors

Several studies have proposed that permeability factors may play a crucial role in the pathogenesis of certain podocyte diseases. This hypothesis is sustained by several observations. For instance, patients with primary FSGS develop recurrence shortly after kidney transplantation, suggesting the presence of circulating factors that may drive podocyte injury. Furthermore, these patients often experience significant reduction in proteinuria after plasmapheresis, which provides additional evidence for the existence of a circulating permeability factor. Also, it was reported that murine models developed proteinuria following the injection of sera from patients with FSGS [23]. These findings collectively suggest that permeability factors could play a crucial role in driving podocyte injury. Hemopexin [24], circulating ANGPTL4 [25], suPAR [26], CLCF1 [27], and Apolipoprotein A-Ib (ApoA-Ib) [28,29] have been suggested as potential permeability factors. Research has also identified several intracellular pathways involved in the action of these factors. For instance, CLCF1 has been shown to activate intracellular cascades such as Janus Kinase 2 (JAK2) and Signal Transducer and Activator of Transcription 3 (STAT3), which ultimately affect cytoskeleton polymerization and can interfere with ß1 integrins binding at the FA level [30]. Apolipoprotein A-Ib is a form of Apolipoprotein A-I that was found to be associated with recurrence of FSGS in kidney-transplant patients; the pathogenic pathway is unknown, yet one proposed mechanism suggests an abnormal activity of a protease, producing both ApoA-Ib and podocyte dysfunction [31].

### 2.4. Immune-Mediated Injury

Immune mechanisms are an important component undermining podocyte integrity, and several distinct pathways may be involved in this process (Figure 2):

- T lymphocytes have long been suspected of being involved in podocyte dysfunction, particularly in MCD. Podocytes express major histocompatibility factor I/II proteins which are involved in immune recognition, as well as B7-1 (CD80), which are T cell co-stimulatory molecules. These influence the activation of lymphocytes, trigger imbalance between effector and regulatory T lymphocytes, as well as interleukin (IL) and other cytokine synthesis by T lymphocytes. For example, IL-17 synthesis by CD17 lymphocytes, with subsequent TNF-α-induced inflammatory cascade [32] and inflammatory responses [33], are all consequences of podocyte/immune cell interactions. More recently, the role of B lymphocyte-driven mechanisms in podocyte injury has gained attention. Podocytes also express B7-2 (CD86), a co-stimulatory molecule that plays a key role in the activation of B lymphocytes and antigen-presenting cells [34]. Recent studies have convincingly documented antibody-mediated podocyte injury, providing evidence for the pathogenic role of nephrin antibodies in steroid-responsive MCD [35]. In addition to nephrin, several antibodies, such as those against annexin A2 [36], or other components of the cytoskeleton [37] have been suggested as etiological factors in certain subsets of podocyte diseases.
- Podocytes also express recognition receptors such as TLRs, which can recognize pathogen-associated molecular patterns (PAMPs), including DNA/RNA fragments. Upon recognition, TLRs activate intracellular pathways that ultimately lead to generation of reactive oxygen species (ROS), mitochondrial stress, and endoplasmic reticulum stress [38]. Additionally, podocytes can respond to metabolic stimuli (e.g., hyperglycemia) and toxic environment stimuli (e.g., puromycin aminoxide toxicity). Such stimuli lead to the activation of intracellular pathways such as NF-κB or MAPK, triggering the production of inflammatory cytokines (IL-1 and IL-18) and TGF-ß, increase oxidative stress, and activate the inflammasome, ultimately resulting in podocyte damage and apoptosis [34,39,40].
- Podocytes are increasingly recognized for their role in regulating the complement system. They are able to synthetize components of the complement system including C1q, C1r, C2, C3, C3a receptor (C3aR), C5a receptor (C5aR), and C7, as well as their inhibitors such as CD47, CD55, CD59, soluble complement factor I (CFI), and complement factor H (CFH). This enables podocytes to precisely regulate inflammatory responses triggered by complement, thereby supporting glomerular homeostasis and reducing damage caused by complement activation. However, podocytes can also become targets of complement-mediated injury under certain instances. For example, in an elegant experimental model of adriamycin-induced podocyte injury, the lack of a C3 convertase inhibitor (CD55) exacerbated podocyte injury and proteinuria, suggesting that complement regulation is essential for podocyte protection [41]. Moreover, podocytes are heavily dependent on factor H protection against excessive complement activation, as complement factors can leak into the GBM, especially under proteinuric conditions. Factor H is able to inactivate them by N terminal binding while being anchored through the C terminal fragment to components of the GBM. Additionally, studies have shown that puromycin/immunotoxin-induced podocyte injury leads to increased factor H expression in podocytes, which correlates with the clearance of subendothelial immune complex deposits [42]. Finally, recent evidence suggests that the activation of the lectin pathway of the complement system may contribute to the pathogenesis of FSGS [43].
- Recent studies have increasingly recognized that sub-lytic levels of the membrane attack complex (MAC) can induce podocyte injury without causing cell death. When internalized, excess MAC can influence calcium (Ca^2+^) entry into the cell, leading to a cascade of downstream effects. One major consequence is the activation of the NF-κB-mediated inflammatory response, which can play a critical role in podocyte dysfunction. Additionally, excessive calcium influx can dysregulate oxidative stress pathways and cause endoplasmic reticulum and mitochondrial stress [44].

### 2.5. Toxic-Induced Podocyte Damage

Puromycin and adryamicin are among the most commonly used substances to induce FSGS and MCD in murine models. Both puromycin aminonucleoside nephrosis and adriamycin-induced nephropathy have a highly proteinuric early phase, resembling the MCD model characterized by complete FPE. This is followed by the progressive development of segmental sclerosis lesions, resembling FSGS. In humans, one of the most frequent toxic causes of podocyte injury is the use of anabolic-androgenic steroids [45], which disrupts SD structure through excess complement activation [43] and increases the production of profibrotic cytokines (TGF-ß) and inflammation [46].

### 2.6. Mechanical Stress

As previously mentioned, an important source of podocyte dysfunction is mechanical stress. Podocytes play a crucial role in maintaining glomerular filtration, and their intricate cytoskeletal structure is continuously adapting to the environmental changes sensed by FA and SD complexes. However, under conditions of hemodynamic stress and/or structural deficiencies, mechanical forces can overwhelm the adaptability of the podocyte, resulting in FPE or even podocyte detachment. Increased glomerular capillary pressure results in the enlargement of capillaries and causes stress to foot processes, enabling elongation and/or shape modification. Glomerular hypertrophy, a universal adaptative response mechanism after kidney injury, is primarily mediated through the activation of tubulo-glomerular feedback and serves as a major driver of mechanical podocyte injury [10,44]. Recent studies have identified several pathways involved in regulating cytoskeletal conformation under mechanical stress. For example, the interaction of suPAR leads to the increased expression of αvß3 B integrin, especially in patients with the APOL1 gene variant. This interaction activates Rho GTPases like CDC42, RhoA, and RAC1, contributing to cytoskeleton dysfunction. Additionally, tensile stress is transmitted by ß integrins to the erythrocyte membrane protein band 4.1 like 5 (EPB41L5), and through multiple signaling molecules (e.g., YAP), it modulates Rho-GTPase activity. The activation of TRPC channels by angiotensin receptors or mechanical stress leads to intracellular calcium influx, which, in turn, activates calcineurin, resulting in synaptopodin degradation. Additionally, increased intracellular calcium promotes the production of reactive oxygen species and mitochondrial and endoplasmic reticulum stress, and can lead to NF-κB and NFAT pathway activation, leading to inflammation and further podocyte damage. Moreover, RhoA1, Rac1, and CDC42 can be directly regulated by the activity of TRPC channels and other ligands [10].

Given the fact that proteinuria and FPE in podocyte diseases are primarily the result of cytoskeleton rearrangements but triggered by different etiologies and mediated by various intracellular pathways, we propose that a classification system based on etiopathogenetic mechanisms—rather than morphology—would be more adequate for this spectrum of diseases. This approach could also potentially lead to more precise diagnostic and therapeutic strategies, ultimately improving patient outcomes by targeting the underlying molecular pathways driving podocyte dysfunction.

## 3. From Pathogenesis to Therapeutic Approach

The choice of therapy for podocyte diseases should be tailored to the pathogenetic mechanism underlying the condition, as the effectiveness of the therapeutic approaches is often contingent on the underlying cause.

For instance, steroid resistance occurs in 8–25% of MCD and FSGS cases, with resistance being more common in FSGS than in MCD. Additionally, steroid resistance is the most important prognostic factor for kidney failure [47].

Corticosteroids exert their effects by binding to steroid receptors on podocytes, leading to the suppression of proinflammatory mechanisms including the synthesis of ILs and TNF-α [48]. Furthermore, corticosteroids help stabilize actin filaments, prevent apoptosis, and increase the expression of the nephrin gene, thereby stabilizing the SD structure [49]. In cases of corticosteroid resistance, cyclophosphamide may be used as it enhances CD4+ T-cell activation, modulates the TLR/MyD88/MAPK pathway, and reduces Th17 generation, thus exerting immunosuppressive effects that help manage podocyte injury [50].

Calcineurin inhibitors, such as ciclosporin and tacrolimus, not only act as immunosuppressants, but also directly stabilize the cytoskeleton in podocytes primarily by inhibiting synaptopodin degradation, regulating podocyte dynamics, and reducing cell apoptosis [51,52,53].

With the discovery of anti-nephrin antibodies (and other antibodies targeting the cytoskeleton) as a culprit in the pathogenesis of MCD/FSGS, B-cell suppression using rituximab has emerged as an interesting therapeutic option, allowing for more prolonged remissions, and is highlighted as a promising approach in a recent position paper of the Immunology European Renal Association Working Group [47]. However, several other potential therapies are under investigation that are directed against some of the pathogenic mechanisms mentioned. These include anti-TNFα therapy, B-cell targeted therapy, and the blockade of TRPC 5/6 channels to regulate intracellular calcium entry. Also, targeting intracellular proinflammatory pathways is a future strategy being explored [47]. Complement inhibition strategies in the current effervescent era of anti-complement therapy validated in different glomerular diseases could also be an option for research, but more data and study trials are required [54].

The current management and diagnosis strategy for MCD and FSGS is based on kidney biopsy. Putting aside its invasive nature, the main shortcoming of the current approach is its inability to identify the underlying etiology and pathophysiological mechanisms at play, and hence its inability to guide a targeted treatment approach. Additionally, biopsy findings often fail to differentiate clearly between primary (amenable to immunosuppression) and secondary/genetic forms of FSGS [5]. Hence, reliance on histology solely must be reevaluated.

A more personalized and optimized approach is clearly needed. Such approaches should be guided by a deeper understanding of the specific molecular mechanisms underlying each patient’s condition, enabling more precise diagnosis and tailored treatment options.

## 4. Advancements in Biomarker Discovery: Insights from Proteomic Approaches

A potential way to guide therapy is the identification of the pathogenic pathway involved by means of biomarkers. This approach could not only help to pinpoint the underlying mechanisms involved in individual cases, but also aid in selecting patients included in clinical trials of targeted therapy in order to maximize the chance of discovering efficient treatment strategies.

Therefore, research on biofluid (urine and plasma) proteomic biomarkers that can both identify the disease (MCD/FSGS) and point to a specific pathogenic pathway—regardless of the morphological form of podocyte injury—offers a promising approach. Furthermore, these biomarkers could potentially predict the response to treatment. Several attempts have been made over the past decades to identify panels of biomarkers that allow for the non-invasive characterization of MCD/FSGS in both experimental and human diseases. These studies build upon previous findings from genomics and transcriptomics, which have offered insights into podocyte disease mechanisms. However, we do not dwell on these data as they are beyond the scope of the current review, especially since increased gene expression does not necessarily result in protein translation or directly influence disease phenotype.

The main advantage of biofluid proteomics is represented by its non-invasive nature. Moreover, urinary proteomics has the potential to directly reflect modifications within the kidney. However, there are also potential downsides due to the nature of the disease; namely, plasma-filtered proteins in these heavily proteinuric conditions may affect urinary findings or, conversely, the accumulation of plasma proteins secondary to decreased glomerular filtration rate can confound the findings. A summary of key proteomic studies and their main findings is provided in Table 1 and discussed below.

### 4.1. MCD/FSGS Versus Other Nephrotic Syndromes/Healthy Controls

One potential use of urinary and plasma proteomics is to identify a panel of proteins that are able to non-invasively differentiate primary podocytopathies (MCD vs. FSGS) from other nephrotic conditions or healthy controls. When compared to controls, the urinary proteomic profile of the murine model of FSGS showed upregulated proteins involved in hemodynamic disturbance, apoptosis, ECM protein deposition, and sclerosis including COL4A1, ECM-1, KLK, KNG1 precursor, ANXA1, CDH1, and ADAM32 [55]. Additionally, molecules involved in oxidative stress were differentially expressed in the urine of children with idiopathic nephrotic syndrome compared to controls [56]. Another study showed that albumin fragments and α1-antitrypsin can differentiate MCD/FSGS/membranous glomerulopathy (MN) from controls [57]. In a separate study, a comparison of urine samples from 11 patients with FSGS, 6 patients with IgA nephropathy (IgAN), and 8 healthy controls revealed that CD59, CD44, IBP7, Robo4, and DPEP1 were the most significantly differentially expressed proteins for FSGS [58]. In another study, a panel of 65 plasma proteins was shown to be differentially expressed in MCD versus MN or healthy controls, with members of the serpin family identified as a signature of MCD [59]. Elevated urinary SERPINA1, alongside CD14 and C9, was suggested to be associated with primary podocyte diseases and proposed as a signature panel for MCD [60]. In the same line of research, RBP4 and SH3BGRL3 were found to differentiate between MCD and diabetic nephropathy [61]. Moreover, downregulated urinary UMOD and upregulated SERPINA1 were found to differentiate MCD and FSGS from healthy controls [62].

### 4.2. MCD Versus FSGS

A complementary approach aims to differentiate MCD from FSGS, and several contributions are noted in this regard. In a study by Perez et al. [63], using the MALDI-TOF proteomic technique, discovered that urinary proteinsUMOD, SERPINA1, B2M, and ALB can help differentiate MCD from FSGS. In a distinct study, it was observed that SERPINA1, TF, HTN3, and MRPL17 levels were reduced in FSGS when compared to MCD, whereas CALB2 levels were elevated [64]. Additionally, Apolipoprotein A1, Alpha 2 macroglobulin (A2M), and ORM2 could differentiate steroid-resistant MCD from steroid-resistant FSGS. In the same study, significantly higher values of retinol-binding protein 4 (RBP4) were observed in patients with FSGS compared to those with MCD [66]. Another study identified ApoA4, HPX, VTN, GSN, and components of the complement system (C4b and factors B and I), along with retinol- and vitamin D-binding proteins, as proteins able to differentiate MCD from FSGS (differentiated according to the severity of the disease and response to treatment [65]). Notably, in the same study, patients classified with the “severe” form of FSGS exhibited increased urinary proteins including components of the MAC (C8a and C9) and downregulation of the protective factor CD59.

### 4.3. Primary Versus Secondary FSGS

Another interesting and contributive approach is aimed at differentiating primary from secondary FSGS. This is important from a clinical and therapeutic point of view as the therapeutic approach is fundamentally different. In this regard, Hellin et al. [68] described three very low-molecular-weight albumin fragments which were present in the plasma of patients with genetic FSGS as opposed to idiopathic FSGS, offering a potential biomarker for distinguishing between these two etiologies. Furthermore, a comprehensive study has described a panel of 93 proteins, including upregulated collagen fragments, SERPINA1, UBE3A, RNF146, complement C3, and PLG, as well as a downregulated fragment of PIGR, which collectively serve as biomarkers to differentiate primary from secondary FSGS [67].

### 4.4. Prognosis and Response to Treatment

The proteomic characterization of biofluids can be used as a non-invasive approach for predicting response to treatment and prognosis in podocyte diseases. There are several attempts published in this area, with one notable investigation using a murine model of adriamycin-induced FSGS. This study showed specific trends in the urine proteome pattern as disease progressed: the upregulation of proteins such as AFM and CP and the downregulation of CDH2 and ACAN were observed throughout the course of the disease. In contrast, distinct trends for FETUB and B2M were noted in different stages of FSGS in mice [69]. Several studies have identified differentially expressed proteins that may serve as biomarkers to differentiate between subtypes of nephrotic syndrome or predict prognosis. For instance, differentially expressed proteins such as rpmF, SAMDC1, FKBP1A, and rpsK were claimed to differentiate between steroid-sensitive nephrotic syndrome and steroid-resistant nephrotic syndrome [70]. Also, decreased urinary CDH1and CDH3 were observed in patients in remission from nephrotic syndrome, suggesting their potential contribution to pathogenesis [72]. Kalantari et al. [71] also identified a panel of urinary prognostic biomarkers in patients with FSGS, with DNASE2 and HP showing the greatest fold change in terms of overrepresentation and underrepresentation in FSGS patients with the best and worse prognosis. A particular form of Apolipoprotein, Apo-A1b, was first increased in the urine of FSGS patients with relapsing disease but not in genetic or non-relapsing forms [29]. Chhuon et al. [28] shed light on some potential mechanisms involved in FSGS recurrence, by comparing plasma proteomic findings (including soluble proteins and extracellular vesicle proteomic profile) of post-transplant recurrent FSGS patients to non-INS patients and healthy controls: upregulated proteins were involved in neutrophil degranulation and downregulated proteins involved in platelet degranulation and lipid-binding (including APOA1). In the same study, proteomic findings of podocytes exposed to the plasma of patients with recurrent FSGS revealed the dysregulation of mTOR pathway and significant differences in proteins involved in cytoskeleton organization. Another study reported APOA1 and MXRA8 as the most significant proteins with the highest fold-change differentiating steroid-resistant and steroid-sensitive FSGS [74]. Additionally, alpha-1-B glycoprotein was found to be associated with steroid-resistant FSGS [73].

The current biomarker research snapshot for podocyte disease can seem confusing at this point. Differences can partly be explained by the targeted patient population and by the differences in proteomic technique. Also, the untargeted approach to biofluid biomarkers can be influenced by pathogenesis, proteinuria, and/or reduced renal function; all these factors need to be addressed in order to improve prediction by biomarkers.

## 5. Proteomic Biomarkers: The Way Forward?

Proteomic analysis has changed over time: as more advanced technologies, such as high-resolution mass spectrometry and refined protein quantification methods, continue to evolve, they provide greater sensitivity and accuracy in detecting low-abundance proteins and post-translational modifications. These advancements enable a more comprehensive and detailed analysis of biofluids, increasing the potential to identify novel biomarkers. Accordingly, the success of biomarker discovery is in enhancing the sensitivity and specificity of proteomic analyses. Effective sample preparation is critical in improving the detection of low-abundance proteins. An option for biological fluids would be affinity-based depletion tools which selectively remove high-abundance proteins; in tissue samples, Laser Capture Microdissection (LCM) can be a pertinent option. LCM enables the isolation of specific tissue regions and structures (such as the isolation of glomeruli), improving both the sensitivity and tissue specificity, thus providing insights into localized disease processes.

Bead-based sample preparation methods have gained attention (SP3 approach—Hughes), which is an excellent option for scenarios involving low amounts of samples. In the context of proteomic studies on kidney disease, a combination of the above-mentioned sample preparation strategies was employed by Höhne et al. [78]. The authors proposed an optimized sample preparation protocol for the analysis of the proteome of a single glomerulus, enabling them to quantify the proteomes in kidney nephron segments consisting of as few as 200 cells and monitor podocyte marker proteins from as few as 80 podocytes per glomerulus independently of antibodies.

The need to analyze low sample amounts underscores the growing requirement to dissect complex biological systems into their individual components, particularly individual cells, in order to dissect their intrinsic heterogeneity. Advancement in techniques has allowed single-cell proteome characterization, which was able to achieve a depth profile of thousands of proteins per cell [79]. One of the most recent technique developments resulted in deep visual proteomics, which merges artificial intelligence image analysis, automated laser microdissection, and high-sensitivity mass spectrometry while maintaining the spatial information of the single-cell proteomic findings [80]. While single-cell proteomics in renal tissues is still in its early stages, there have been promising advancements enhancing all aspects of MS-based proteomics analytical framework—from sample preparation to MS data acquisition and processing.

An important area of proteomics is the study of post-translational modifications (PTMs). These modifications (phosphorylation, glycosylation, etc.) play a crucial role in regulating protein function, stability, localization, and interactions and, subsequently, can dramatically impact cellular processes. In the context of kidney diseases, these modifications have been found to be integral to pathogenesis, as they are associated with cellular responses to injury, fibrosis, and inflammation in renal tissues. The authors refer the reader to the excellent, comprehensive overview of Liu et al. [81] which details the molecular mechanisms by which PTMs contribute to the pathogenesis of renal diseases. In FSGS, the study by Chhuon et al. [28] provides critical insights into the early signaling events that occur in podocytes after exposure to plasma from rFSGS patients. The authors highlighted that, following the exposure of podocytes to rFSGS plasma, several phosphoproteins involved in cytoskeleton rearrangement and mTOR activation were significantly modified. The ability to analyze these PTMs at a cellular level, particularly through advancements in single-cell proteomics, offers a promising avenue for understanding the complexities of kidney disorders.

Bioinformatic tools have significantly enhanced proteomic study outcomes by enabling the identification of connections at the pathway level, improving the interpretation of complex biological data, and facilitating the discovery of key biomarkers and pathways involved in various diseases. These tools also aid in visualizing large-scale data generated from proteomic experiments and in integrating results from other omics fields while covering several stages of analysis: from MS raw data analysis and protein identification and annotation to the post-processing steps, including functional and pathway analysis, protein–protein interaction networks, statistical analysis and validation, integration with other omics data, and data visualization. While the increased availability of computational resources has made these tools more accessible, it has also made it easier to make mistakes, leading to inflated or misleading results. This issue is compounded by the lack of standardized guidelines in the field regardless of the level at which these tools are applied, further increasing the potential for errors.

Furthermore, evolving data analysis techniques, including machine learning algorithms, may contribute to more precise interpretation of complex proteomic data, enabling better classification and prediction of disease subtypes and treatment responses. Another direction is the integration of multi-omics approaches, such as mass spectrometry-based metabolomics, as it can provide a more holistic understanding of disease pathways. These advancements bring us closer to personalized medicine in podocyte diseases, where tailored therapeutic strategies can be developed based on individual biomarker profiles and underlying pathogenic mechanisms. These advancements are one of the reasons for which proteomic research holds significant promise as a way towards personalized medicine in this field, pending several amendments.

In our view, it is difficult to assess the value of a broad biomarker panel or an untargeted biofluid proteomic approach applied indiscriminately to the entire spectrum of MCD/FSGS. It would be of incomparably greater value if the proposed biomarkers were directly linked to a specific pathogenic pathway, hence enabling the identification of a subset of patients with a defined mechanism for podocyte injury, potentially amenable to a specific therapeutic approach.

Based on pathogenesis, some contributions of targeted proteomics approaches can be listed:

- Complement activation has emerged as an important mechanism in the pathogenesis of FSGS and is strongly associated with more severe morphological injury and poorer prognosis. Decreased plasma C3 levels were associated with loss of kidney function and, more importantly, proteinuria and tubulointerstitial injury, as well as a progressive renal disease in FSGS [82]. Furthermore, both plasma and urine complement proteins, including MAC, correlate inversely with kidney function and directly with proteinuria and histologic findings in FSGS [83,84]. Recent reports have indicated that urinary C5a and MAC may serve as useful biomarkers for distinguishing FSGS from MCD [85].
- B lymphocyte-mediated injury, as evidenced by the presence of nephrin antibodies, annexin antibodies, and other antibodies [36,37,75], has been identified as a key contributor to disease in certain subsets of patients within the MCD/FSGS spectrum.
- T lymphocyte-mediated mechanisms have been identified through the increased CD80 levels in the urine of nephrotic MCD patients, a marker not observed in patients in remission from MCD or in FSGS patients [86]. This suggests that CD80 may serve as a potential biomarker for active disease and could play a role in the pathogenesis of nephrotic syndrome in certain patient subsets.
- Proinflammatory and profibrotic pathways can be reflected by elevated urinary TGF-ß levels, which are reported to be higher in FSGS compared to MCD [87].

A step forward in identifying the pathogenic mechanisms involved and developing more accurate biofluid biomarkers is the untargeted proteomic glomerular study. In recent decades, the advancement of proteomics techniques, coupled with the ability to separate glomerular tissue from formalin-fixed, paraffin-embedded biopsies, has led to tremendous progress in the characterization of several glomerular diseases, like MN, C3 glomerulonephritis, and amyloidosis. These studies have, in many cases, resulted in the reclassification of diseases (membranoproliferative glomerulonephritis and amyloidosis) or prompted a reevaluation of diagnosis and management in MN [1]. However, attempts to characterize the spectrum of MCD/FSGS through these methods are surprisingly scarce. One study, published in abstract form, suggests that secondary FSGS is associated with upregulated complement pathways, while the primary form of FSGS shows upregulated proteins involved in cell-to-cell and matrix adhesion compared to MCD [88]. Furthermore, one study performed proteomic analysis on whole FFPE renal tissue and discovered upregulated LAMP1 and ACSL4 in pediatric resistant FSGS, and their finding also support complement activation in FSGS [89]. Upregulated complement alongside fibrinogen pathway and protease inhibitors (SERPINA1 and SERPINA3) were also described in a different study by the characterization of ECM in a murine model of FSGS [90].

Another study suggested that complement-regulating proteins, such as CD59, were substantially decreased in MCD when compared to other glomerular diseases known for complement-related inflammation (MN or IgAN) [75]. Our own research identified 58 significantly different proteins when comparing MCD, FSGS, and healthy controls; the pathway analysis of these proteins was associated with cytoskeleton dynamics and nephrin interactions [76]. Furthermore, we identified ANXA2 as one of the significantly increased proteins in MCD compared to controls [12].

**Figure 4:**
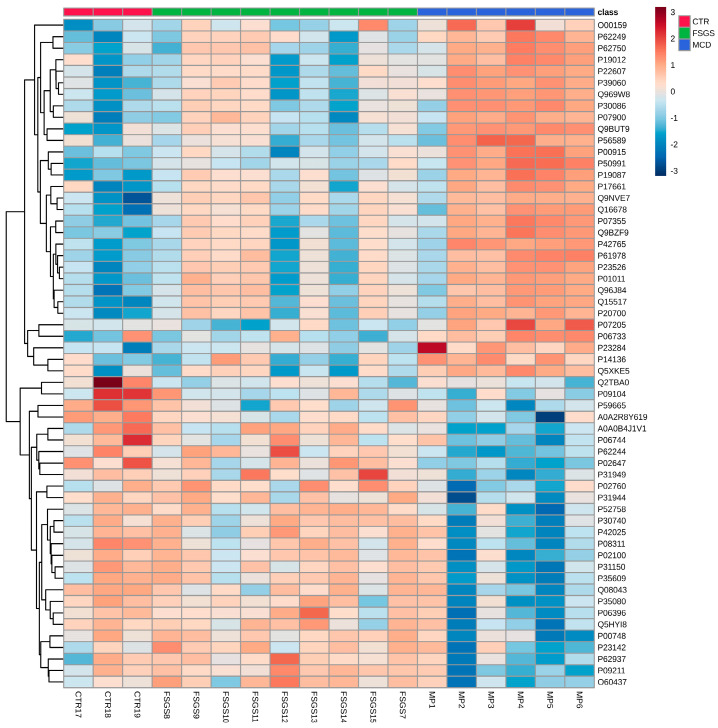
Differentially expressed proteomic signatures of patients with FSGS, patients with MCD, and healthy controls. Reprinted with permission from Bărar et al. [76].

We believe that efforts focusing on tissue proteomics in the FSFS/MCD spectrum has to be amplified for at least two reasons. First, given the considerable heterogeneity of pathophysiological mechanisms in MCD/FSGS, proteomic findings can vary significantly from one patient to another. In our own experience, differentially expressed proteins can differ within the FSGS or MCD groups, which is eventually a reason to reclassify patients within the MCD/FSGS spectrum. For instance, in one of our previous findings, the proteomics profiles of certain MCD patients were more closely related to controls, while others resembled FSGS profiles (as shown in Figure 4, which presents a heatmap of differentially expressed proteins in MCD/FSGS and healthy controls [76]). To enhance the value of proteomic findings, more uniform pathogenesis-based cohorts with larger numbers of patients are needed. Glomerular proteomic studies should be able to ultimately distinguish between specific pathways of disease. This is elegantly illustrated in a study which shows that collapsing forms of FSGS are driven by enzymes such as cathepsin B and cathepsin C, derived from activated parietal cells infiltrating glomerular tufts that result in distinctive patterns in matrix production and/or degradation, not found in non-collapsing forms. The parietal epithelial cell signature in collapsing FSGS is illustrated by an increase in specific proteins, such as annexin A3, alongside cathepsin B and cathepsin C [77].

Second, once one or more significantly upregulated proteins have been identified in glomerular tissue, an attempt can be made to measure them in urine or plasma. Of course, tissue, plasma, and urine have different proteomic signatures as they are different compartments and an upregulated tissue protein is not necessarily found in urine (and/or plasma). However, certain proteins with possible pathogenic roles could derive from plasma (e.g., cytokines, enzymes, and permeability factors) and, more importantly, urine proteins can reflect shedding of tissue proteins. For example, C4d is found in glomeruli of FSGS even before sclerosis develops [43], and increased urinary and plasma C5b-9 and C5a in FSGS have been reported [84,85]. Urinary nephrin and podocalyxin are early markers of proteinuric glomerular diseases, reflecting podocyte injury [91,92].

Biofluid-directed proteomic studies offer the advantage of being non-invasive, but, as mentioned, they can be influenced by several factors: renal function can result in an increase in plasma proteins and proteinuria, and influence the abundance of urinary proteins, not necessarily reflecting pathogenic process within the glomeruli (accidental findings), and the tubular compartment can further confound results (protein reabsorption or tubular source of proteins). The complexity of the relation of tissue proteomics to urinary findings is reflected in the aforementioned study by Merchant et al. [77], in which some, but not all, of the urinary proteins found to be increased in collapsing FSGS are correlated to the tissue abundance of proteins. However, the value of biofluid proteomic findings would be significantly enhanced if studied within a prospective cohort guided by tissue proteomics findings.

**Figure 5:**
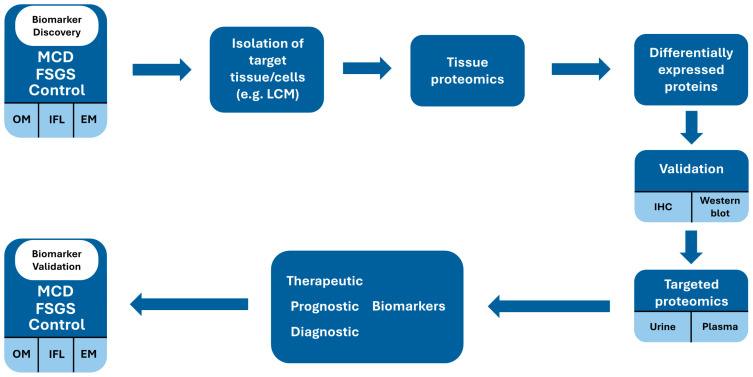
Proposed approach for biomarker development.

Such biomarkers could be evaluated at the time of first presentation and repeated during remission and during relapse in order to certify that they are influenced by the pathogenic process of podocyte injury and are not an accidental finding. A similar approach would determine whether they also have a prognostic value in terms of predicting response to therapy or relapsing course. This approach would enhance the clinical applicability and accuracy of biofluid biomarkers for monitoring and choice of therapy. A potential workflow is suggested in Figure 5.

## 6. Concluding Remarks/Future Perspectives

The spectrum of MCD/FSGS needs urgent reassessment based on pathogenetic mechanisms in order to allow personized treatment and improve prognosis. A merely histological approach is insufficient whereas increasingly refined proteomic techniques might be an important tool for a personalized, fine-tuned diagnostic and therapeutic approach.

Our proposed approach in biomarker research aims to transition from tissue proteomics, which, as mentioned before, most closely reflects the underlying pathological mechanisms and structural changes within the podocyte diseases, to urine or plasma protein profiling.

In this way, a patient or subgroup of patients exhibiting the same pathophysiology of podocyte injury, as identified by tissue proteomic profiling, could potentially be characterized by biofluid biomarker approaches. This would offer significant value for non-invasive diagnostic perspectives and, most importantly, for prognostic evaluation and therapeutic decision-making.

## Figures and Tables

**Table 1:** Current proteomic research results relevant to podocyte diseases and their main findings.

Study	Groups	Tissue/Biofluid	Method	Significance
Shui et al. [55], 2008	(Murine model) FSGS and healthy controls	Urine	2DE, MALDI-TOF MS	FSGS—upregulated proteins involved hemodynamic disturbance, apoptosis, ECM protein deposition, and sclerosis (COL4A1, ECM-1, KLK, KNG1 precursor, ANXA1, CDH1, and ADAM32)
Sedic et al. [56], 2014	12 INS, 12 healthy controls	Urine	LC-MS	INS—74 proteins upregulated, 9 potential biomarkers Oxidative stress may be a pathogenic mechanism
Candiano et al. [57], 2006	10 MCD, 7 FSGS 6 MN, 10 healthy controls	Urine/Plasma	2DE, MALDI-TOF/MS	MCD/FSGS/MN—upregulated Albumin fragments and SERPINA1 in urine and plasma compared to healthy controls
Nafar et al. [58], 2014	11 FSGS,6 IgAN, 8 healthy controls	Urine	nano-LC/MS	FSGS—upregulated CD59, CD44, IBP7, Robo4, and DPEP1
Muruve et al. [59], 2022	15 MCD, 37 MN, 20 healthy controls	Plasma	SOMAscan	MCD—70-protein signature; serpin family proteins downregulated compared to MN/healthy controls (SERPINA10, SERPINA4, SERPINC1, SERPINF2, and SERPINF1); complement system and coagulation pathway downregulated MCD—upregulated immune and growth factor signaling proteins (STAT1, STAT3, CD40LG, and FGF16); carbohydrate and lipid metabolism (GAPDH, GSK3A/B, PKM2, HK2, CHST6, LRP1B, APOE, and APOA1) compared to MN/healthy controls
Choi et al. [60], 2017	Discovery cohort: 4 MCD, 4 FSGS, 4 MN, 4 healthy controls Validation cohort: 13 MCD, 5 FSGS, 26 MN, 9 IgAN, 8 healthy controls	Urine	SDS-PAGE, LC-MS	MCD—upregulated CD14, C9, and SERPINA1 FSGS—upregulated CDH26, RNASE1, and DIS3L
Araumi et al. [61], 2021	14 MCD, 11 DN, 23 MN	Urine/Plasma	nano-LC/MS	DN—upregulated urinary RBP4 and SH3BGRL3 compared to MCD
Navarro-Muñoz et al. [62], 2012	9 FSGS, 3 MCD, 9 IgAN, 6 MN, 7 healthy controls	Urine	HPLC-MS/MS	MCD/FSGS—upregulated SERPINA1 and downregulated UMOD compared to healthy controls
Perez et al. [63], 2014	22 FSGS, 22 MCD	Urine	MALDI-TOF MS	UMOD and SERPINA1 can differentiate between FSGS and MCD
Perez et al. [64], 2017	25 FSGS, 24 MCD	Urine	2D-DIGE; MALDI-TOF MS	MCD—upregulated SERPINA1, PTAFR, CCNY, TF, HTN3, and MRPL17 FSGS—upregulated CALB2
Chebotareva et al. [65], 2022	30 FSGS, 9 MCD	Urine	LC-MS	“Severe” FSGS vs. “mild” FSGS/MCD—upregulated complement activity (C4b, C9, CFB, and CFI); upregulated podocyte damage (VTN, HPX, GSN, and APOA1); upregulated ECM accumulation (CST3, DBP, RBP4, AHSG, SERPING1, LUM, and CLU)
Suresh et al. [66], 2016	55 INS: 5 SRNS MCD, 5 SRNS FSGS, 2 SRNS MN	Urine	iTRAQ LC/MS	SRNS FSGS vs. SRNS MCD—upregulated A2M and ORM2
Catanese et al. [67], 2023	19 primary FSGS, 44 secondary FSGS	Urine	CE-MS	Primary FSGS vs. secondary FSGS—upregulated collagen fragments, SERPINA1, UBE3A, RNF146, complement C3, and PLG; Downregulated fragment of PIGR.
Hellin et al. [68], 2009	15 idiopathic FSGS, 11 genetic FSGS	Plasma	2DE, MALDI-TOF MS, Western blot, LC-ES-MS	Three very low-molecular-mass albumin fragments in plasma of patients with genetic FSGS vs. idiopathic FSGS/ healthy controls
Zhao et al. [69], 2014	(Murine model) FSGS	Urine	LC-MS	FSGS—urine protein change pattern in time: upregulation of AFM and CP; downregulation of CDH2 and ACAN; distinct pattern of FETUB and B2M
Bai et al. [70], 2012	9 SRNS, 32 SSNS	Urine	Chip-MS	SRNS—upregulated SAMDC1, FKBP1A, and rpsK SRNS—downregulated rpmF
Kalantari et al. [71], 2014	5 mild FSGS, 5 advanced FSGS	Urine	nano-LC/MS	Mild FSGS—upregulated DNASE2 Advanced FSGS—upregulated HP Complement and coagulation pathways activated in FSGS
Chhuon et al. [28], 2023	4 recurrent FSGS, 4 non-INS controls; post-transplant	Plasma	nano-LC-MS/MS	Recurrent FSGS—upregulated neutrophil degranulation; downregulated platelet degranulation and lipid-binding proteins; dysregulation of mTOR pathway
Lopez-Hellin et al. [29], 2012	6 recurrent FSGS, 34 non-recurrent FSGS; post-transplant	Urine	2DE/MALDI-TOF/LC-ESI-MS/MS	Urinary ApoA-Ib associated with recurrent FSGS
Andersen et al. [72], 2012	4 INS remission/active disease	Plasma/Urine	nano-LC/MS	Active disease—downregulated urinary CDH1, CDH3, KLKB1, HPX
Piyaphanee et al. [73], 2011	19 SRNS, 15 SSNS, 10 healthy controls	Urine	MALDI-TOF/MS	A1BG is associated with SRNS FSGS
Kalantari et al. [74], 2014	6 SS FSGS, 4 SR FSGS	Urine	LC-MS	SS—upregulated APOA1 SR—upregulated MXRA8 Acute inflammatory response was the predominant biological process (CLUS, A1AG2, AACT, and TRFE)
Dong et al. [75], 2023	3 MCD, 11 IgAN, 19 LN. 5 MN, 8 healthy controls	Tissue	LCM + nano-LC-MS/MS	MCD/IgAN vs. LN/MN—downregulated CD59; A2M upregulated in every group but not in MCD; downregulated FLII (regulatory cytoskeleton protein) in glomerular disease versus control
Bărar et al. [76], 2023	6 MCD, 9 FSGS, 3 healthy controls	Tissue	LC-MS/MS	58 significant proteins between the 3 groups
Bărar et al. [12], 2023	5 MCD, 3 healthy controls	Tissue	LC-MS/MS	MCD—upregulated ANXA2 and NID1 MCD—downregulated ZO-1, MYO1C, ITGA3, ACTR3B, and NES
Merchant et al. [77], 2020	Collapsing FSGS 7, NOS-FSGS 6, healthy controls	Tissue/Urine	LC-MS/MS	Collapsing FSGS—distinct pattern of sclerosis compared to other form of FSGS; upregulated cathepsin B and cathepsin C in tissue

Abbreviations used: FSGS—Focal Segmental Glomerulosclerosis; MCD—Minimal Change Disease; MN—Membranous Nephropathy; IgAN—Immunoglobulin A Nephropathy; SRNS—Steroid-Resistant Nephrotic Syndrome; INS—Idiopathic Nephrotic Syndrome; SS—Steroid-Sensitive; SR—Steroid-Resistant; LN—Lupus Nephritis.

## Abbreviations

The following abbreviations are used in this manuscript:

MCD	Minimal change disease
FSGS	Focal segmental glomerulosclerosis
FPE	Foot process effacement
EM	Electron microscopy
LM	Light microscopy
FP	Foot process
SD	Slit diaphragm
FA	Focal adhesion
ECM	Extracellular matrix
GBM	Glomerular basement membrane
TLRs	Toll-like receptors
ILs	Interleukins
MAC	Membrane attack complex
IgAN	IgA nephropathy
MN	Membranous nephropathy
rFSGS	Recurrent focal segmental glomerulosclerosis
MS	Mass spectrometry
